# Combined Anesthesia with Ethyl Chloride and Ether

**Published:** 1904-01

**Authors:** William Pebrin Nicholson

**Affiliations:** Atlanta, Ga.


### COMBINED ANESTHESIA WITH ETHYL CHLORIDE AND ETHER.

BY WILLIAM PEBRIN NICHOLSON, M.D..

ATLANTA, GA.

There is perhaps no question in which every physician is more interested than that of the administration of anesthetics, for no matter where placed, the time must of necessity come when he will be called upon to make use of them.

The selection of an anesthetic is a matter of serious importance, and the method of its administration equally so. There are many factors to be considered in arriving at a conclusion, and circumstances frequently arise where the good of the patient and the future result are determined by the selection.

The ideal anesthetic is that which is safest for the patient; most economical as regards cost; most humane in its administration; speediest in its action; and most free from disagreeable after-effects. It unfortunately happens that in many instances it is not practical to find one combining one of all these features, when it is still the duty of the physician to make the selection, which most nearly attains the end.

In practically every case the choice would have to be made between ether and chloroform, and though chloroform is admittedly the more dangerous of the two, its use, away from the larger centers, is general, largely on account of the fact that its employment is more agreeable to the patient, and its administration does not require any special or expensive apparatus. The old ether cone, which has been the only thing available, is uncertain, slow, and inhumane as well as very wasteful of material. Though many writers have claimed to effect complete anesthesia by ether with an ordinary cone in from five to eight minutes, in the hands of most persons it certainly requires at least three times as long. Hence the satisfactory exhibition of ether has always required some form of apparatus, if we desire to obtain its best and quickest results; and in order to hasten its effects, and at the same time do away with much of its disagreeable suffocation, it has become the established practice of many to begin the anesthesia with some other substance, and complete it with ether. The three most frequently employed for this purpose are chloroform, nitrous oxide gas, and ethyl chloride (Kelene).

With the effects of chloroform all are familiar, its chief claim as a precursor of ether being the fact that it is more agreeable in odor, less irritating to the air passages, and somewhat more speedy in its primary effects; yet we know that many deaths from chloroform have occurred with almost the first inhalation of the drug, and this fact alone makes its use for this purpose practically as dangerous as its employment to the extent of full anesthesis.

The use of nitrous oxide gas as a prelimary step in the exhibition of ether is a most desirable and satisfactory method, and is very constantly used in the hands of many surgeons, including the writer, but is open to the objection that it requires expensive and complicated apparatus, and the supply of gas is not always in reach, and even where it is, the transportation of the receptacle as well as the apparatus is burdensome on account of its weight and bulk. Again, it requires a degree of familiarity with the use of the apparatus, which enforces the employment of an etherizer, who is an expert in its use. These objections apply so forcibly, that the use of this form of anesthesia in the hands of the general practitioner, is practically not to be considered.

In ethyl chloride we have a substance which is easily obtainable, inexpensive, and easily kept. Its administration requires no form of apparatus, except such as is used in administering chloroform, and, as far as known, its use for primary anesthesia is entirely void of danger. This drug has the remarkable effect of destroying all tactile sensation, as well as the sense of smell, without destroying the conciousness of the patient, or in any way affecting his pulse, respiration, or general appearance; and its inhalation is unattended by any stage of excitement or muscular disturbance. It is undoubtedly better in using ether by this method to employ a closed apparatus which absolutely excludes air and preferably one which causes the patient to reinhale the expired air, from a rubber bag, but the use of a close cone, such as is ordinarily employed, will in most cases give almost as satisfactory results, though complete anesthesia requires a little longer time.

In order that the value of this method may be realized and the desired results obtained, I will recite in detail the method employed. An ordinary Esmarch chloroform inhaler is used, and the ethyl chloride administered by spraying from an ordinary Kelene tube, just as chloroform is administered. The spray forms a frost over the cloth covering the inhaler, and is renewed from time to time as this disappears. An assistant usually pinches up the skin of the back of the hand and the patient is asked from time to time if he feels any pain from the pinching, and when he states that he does not the ether inhaler is immediately applied, and usually without any struggle whatever the patient passes rapidly under the influence of the ether, complete anesthesia only requiring from three to five minutes. After complete recovering from anesthesia the patient has no recollection whatever of the beginning of ether, even though answering questions intelligently at the time it begins.

The effect of the administration of Kelene is very different in different individuals, causing very varying subjective sensations. Many patients feel as though they were flying with great velocity through space, others that they are falling rapidly, while some complain of subjective noises, but in no case have I seen any disturbance or alarm from this or any resistance on the part of the patient. In some cases there is a momentary struggle at the beginning of the ether, just as in the use of gas, but it is of short duration, and leaves no impression upon the mind of the patient. One of the greatest advantages of this form of anesthesia is that prolonged nausea after recovery from the ether is almost unknown, there being only one vomiting as the patient comes out from his sleep. Another is the very small amount of ether consumed, as a large amount of that usually employed is expended upon the primary anesthesia, and the small amount employed also renders the patient far less liable to the post-operative dangers of anesthesia.

In conclusion, I would claim that the combined anesthesia by ethyl chloride and ether is the ideal anesthetic in that it is as free from danger as an anesthetic can be; reduces the cost of the an-anesthetic to a minimum; is humane, in that it relieves the patient of all that is most horrible in the taking of an anesthetic, saves the surgeon the time consumed by other methods as well as the expense of apparatus; increases the post-operative comfort of the patient, and lessens in a large degree the danger of complications.

---

SURGEON-GENERAL RIXEY in his annual report recommends that Congressional action be asked for authority to rename the different grades of the medical corps now existing as follows: In place of surgeon-general, surgeon-admiral; and in the other grades medical director to become surgeon-captain; medical inspector, surgeon-commander; surgeon, surgeon - lieutenant - commander; passed assistant surgeon, surgeon-lieutenant; and assistant surgeon, surgeon-lieutenant (junior grade.)

